# Critical roles of extracellular vesicles in periodontal disease and regeneration

**DOI:** 10.1093/stcltm/szae092

**Published:** 2024-12-19

**Authors:** Lin Jing, Hong-Yu Wang, Ning Zhang, Wen-Jie Zhang, Yuzhe Chen, Dao-Kun Deng, Xuan Li, Fa-Ming Chen, Xiao-Tao He

**Affiliations:** State Key Laboratory of Oral & Maxillofacial Reconstruction and Regeneration, National Clinical Research Center for Oral Diseases, Shaanxi International Joint Research Center for Oral Diseases, Department of Periodontology, School of Stomatology, The Fourth Military Medical University, Xi’an 710032, People’s Republic of China; State Key Laboratory of Oral & Maxillofacial Reconstruction and Regeneration, National Clinical Research Center for Oral Diseases, Shaanxi International Joint Research Center for Oral Diseases, Department of Periodontology, School of Stomatology, The Fourth Military Medical University, Xi’an 710032, People’s Republic of China; Cadet Regiment, School of Basic Medical Sciences, Air Force Medical University, Xi’an 710032, People’s Republic of China; State Key Laboratory of Oral & Maxillofacial Reconstruction and Regeneration, National Clinical Research Center for Oral Diseases, Shaanxi International Joint Research Center for Oral Diseases, Department of Periodontology, School of Stomatology, The Fourth Military Medical University, Xi’an 710032, People’s Republic of China; State Key Laboratory of Oral & Maxillofacial Reconstruction and Regeneration, National Clinical Research Center for Oral Diseases, Shaanxi International Joint Research Center for Oral Diseases, Department of Periodontology, School of Stomatology, The Fourth Military Medical University, Xi’an 710032, People’s Republic of China; State Key Laboratory of Oral & Maxillofacial Reconstruction and Regeneration, National Clinical Research Center for Oral Diseases, Shaanxi International Joint Research Center for Oral Diseases, Department of Periodontology, School of Stomatology, The Fourth Military Medical University, Xi’an 710032, People’s Republic of China; State Key Laboratory of Oral & Maxillofacial Reconstruction and Regeneration, National Clinical Research Center for Oral Diseases, Shaanxi International Joint Research Center for Oral Diseases, Department of Periodontology, School of Stomatology, The Fourth Military Medical University, Xi’an 710032, People’s Republic of China; State Key Laboratory of Oral & Maxillofacial Reconstruction and Regeneration, National Clinical Research Center for Oral Diseases, Shaanxi International Joint Research Center for Oral Diseases, Department of Periodontology, School of Stomatology, The Fourth Military Medical University, Xi’an 710032, People’s Republic of China; State Key Laboratory of Oral & Maxillofacial Reconstruction and Regeneration, National Clinical Research Center for Oral Diseases, Shaanxi International Joint Research Center for Oral Diseases, Department of Periodontology, School of Stomatology, The Fourth Military Medical University, Xi’an 710032, People’s Republic of China

**Keywords:** extracellular vesicles, periodontal disease, periodontal regeneration, mesenchymal stem cells, MSC- mesenchymal stem cells-extracellular vesicles

## Abstract

Extracellular vesicles (EVs) are evolutionarily conserved communication mediators that play key roles in the development of periodontal disease as well as in regeneration processes. This concise review first outlines the pathogenic mechanisms through which EVs derived from bacteria lead to the progression of periodontitis, with a focus on the enrichment of virulence factors, the amplification of immune responses, and the induction of bone destruction as key aspects influenced by bacterial EVs. This review aims to elucidate the positive effects of EVs derived from mesenchymal stem cells (MSC-EVs) on periodontal tissue regeneration. In particular, the anti-inflammatory properties of MSC-EVs and their impact on the intricate interplay between MSCs and various immune cells, including macrophages, dendritic cells, and T cells, are described. Moreover, recent advancements regarding the repair-promoting functions of MSC-EVs are detailed, highlighting the mechanisms underlying their ability to promote osteogenesis, cementogenesis, angiogenesis, and the homing of stem cells, thus contributing significantly to periodontal tissue regeneration. Furthermore, this review provides insights into the therapeutic efficacy of MSC-EVs in treating periodontitis within a clinical context. By summarizing the current knowledge, this review aims to provide a comprehensive understanding of how MSC-EVs can be harnessed for the treatment of periodontal diseases. Finally, a discussion is presented on the challenges that lie ahead and the potential practical implications for translating EV-based therapies into clinical practices for the treatment of periodontitis.

## Graphical Abstract

**Graphical Abstract:**
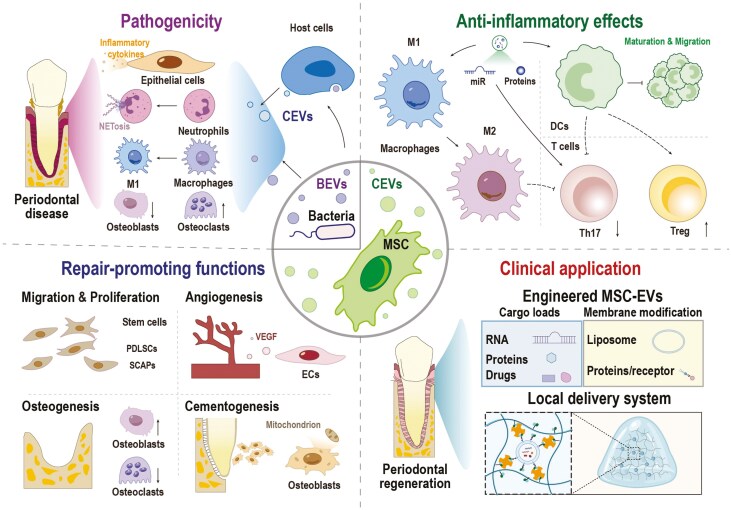

**Significance statement**

This review underscores the critical role of bacterial extracellular vesicles in periodontitis, detailing their pathogenic mechanisms and impact on immune responses and bone resorption. It highlights a novel insight into how bacteria manipulate host cells to enhance inflammation through cellular extracellular vesicles. Furthermore, it emphasizes the therapeutic potential of mesenchymal stem cell-derived extracellular vesicles, showcasing their anti-inflammatory properties and regenerative capabilities in periodontal therapy. By integrating these findings, the article provides a comprehensive understanding of EVs in periodontal disease, paving the way for innovative treatment strategies that could significantly improve therapeutic outcomes.

## Introduction

Periodontitis is one of the most common diseases and affects 61.6% of the population, with 11.2%-12.50% of people experiencing severe manifestations,^1-3^ representing a substantial burden on healthcare systems and resulting in major social and economic challenges. The primary characteristic of periodontitis is the loss of periodontal tissue support, as shown by clinical attachment loss, alveolar bone loss, and the occurrence of periodontal pockets and gingival bleeding. As the leading cause of edentulism and masticatory dysfunction, periodontitis affects the nutrition, facial features, quality of life, and self-esteem of patients and results in major socioeconomic impacts and healthcare costs.^4^ Furthermore, increasing evidence has indicated that periodontitis is critically associated with systemic disorders, such as cardiometabolic, neurodegenerative and autoimmune diseases, and cancer.^5^ Moreover, periodontitis (edentulism) is linked to a greater risk of all-cause and cause-specific mortality.^6^ The current treatment methods for periodontitis include scaling and root planning; however, these strategies can control the development of periodontitis but cannot restore lost periodontal tissues. Guided tissue regeneration is the major method for periodontal regeneration; however, limited regenerative outcomes in the clinic are observed, which is unsatisfactory.^7^

Polymicrobial communities are believed to be the causative agents of periodontitis.^8^ These polymicrobial communities can subvert the host immune response, which can prevent death and further reinforce inflammation in a positive feedback loop. The interactions between bacteria and host cells are crucial in the pathogenesis of periodontal diseases, as noted in several excellent reviews.^9,10^ In these reviews, the roles of cytokines,^11,12^ bacterial virulence factors,^13^ and the direct communication of periodontal pathogens and host immune cells^9^ have been highlighted. Recently, accumulating findings have indicated that extracellular vesicles (EVs) released by bacteria play key roles in intercellular communication. Bacteria-derived extracellular vesicles (BEVs) are nanosized particles enclosed by lipid bilayers, that contain various substances, such as enzymes, toxins, and microbe-associated molecular patterns (MAMPs), that function as virulence factors in the host.^14^ Compared with parental bacteria, BEVs can harbor virulence factors and enter host immune cells, thus resulting in much stronger immune responses and exacerbating inflammation-mediated tissue destruction.^15^ Therefore, summarizing the recent progress in determining the effects of BEVs on the development of periodontal diseases is important.

As evolutionarily conserved communication mediators from bacteria to eukaryotic cells, EVs derived from periodontal cells also contribute to the progression of periodontal diseases and regenerative therapies for periodontal diseases. Owing to the minimal immunogenicity, high safety, and multiple bioactivities of EVs derived from mesenchymal stem cells (MSC-EVs), MSC-EVs are considered the most effective alternatives for mesenchymal stem cells (MSCs); these EVs can exert immunomodulatory effects and promote osteogenesis/cementogenesis/angiogenesis, similar to their parental MSCs. The mechanisms and clinical application potential of MSCs have been extensively described in a series of excellent reviews.^16,17^ Moreover, the therapeutic performance of MSC-EVs can be effectively increased by modifying their cargoes or designing local delivery systems.^18^ Owing to these advantages, EVs have been applied in numerous fields of medicine, including the treatment of neurodegenerative disorders (Alzheimer’s disease, central nervous system), the regeneration of damaged organs (kidney, liver and heart) and tissues(skin and dental pulp), and early diagnosis of cancers and so on.^19,20^ All of these compounds demonstrate great therapeutic effects and application prospects. More importantly, some investigators have also explored the translational prospects of MSC-EVs in the treatment of periodontitis. However, emerging evidence has also indicated that EVs from periodontal cells have pathogenic effects by exacerbating the host immune response and inducing tissue destruction, which cannot be ignored, and little attention has been given to this issue.

Overall, the present review summarizes the latest advancements in the application of EVs as essential mediators involved in periodontal disease and regeneration by mediating intercellular communication among bacteria and bacteria and cell-cell interactions. First, we underscore the importance of EVs in the pathogenesis of periodontitis, encompassing the pathogenic effects of both BEVs and cellular extracellular vesicles (CEVs). We subsequently explored the mechanisms through which CEVs exert anti-inflammatory effects and repair-promoting functions. Finally, we present the translational prospects of EVs in the treatment of periodontitis, along with a description of potential future challenges in this field.

## Pathogenic roles of EVs in the progression of periodontitis

Extracellular vesicles derived from bacteria or cells carry virulence factors and inflammatory mediators, thereby exacerbating inflammation and accelerating tissue destruction during the pathogenesis of periodontitis. On the one hand, virulence factors can be highly enriched in EVs, which can exert even more powerful pathogenic effects than their cells of origin. On the other hand, inflammatory mediators carried by EVs can directly activate the intracellular pattern recognition receptors (PRRs) of cells, leading to an excessive proinflammatory response in cells. In this section, we delve into recent findings on the pathogenic roles of EVs in the development of periodontitis, highlighting their effects on the amplified immune response and accelerated bone resorption.

### Pathogenic roles of BEVs in periodontal homeostasis

**Figure 1.**
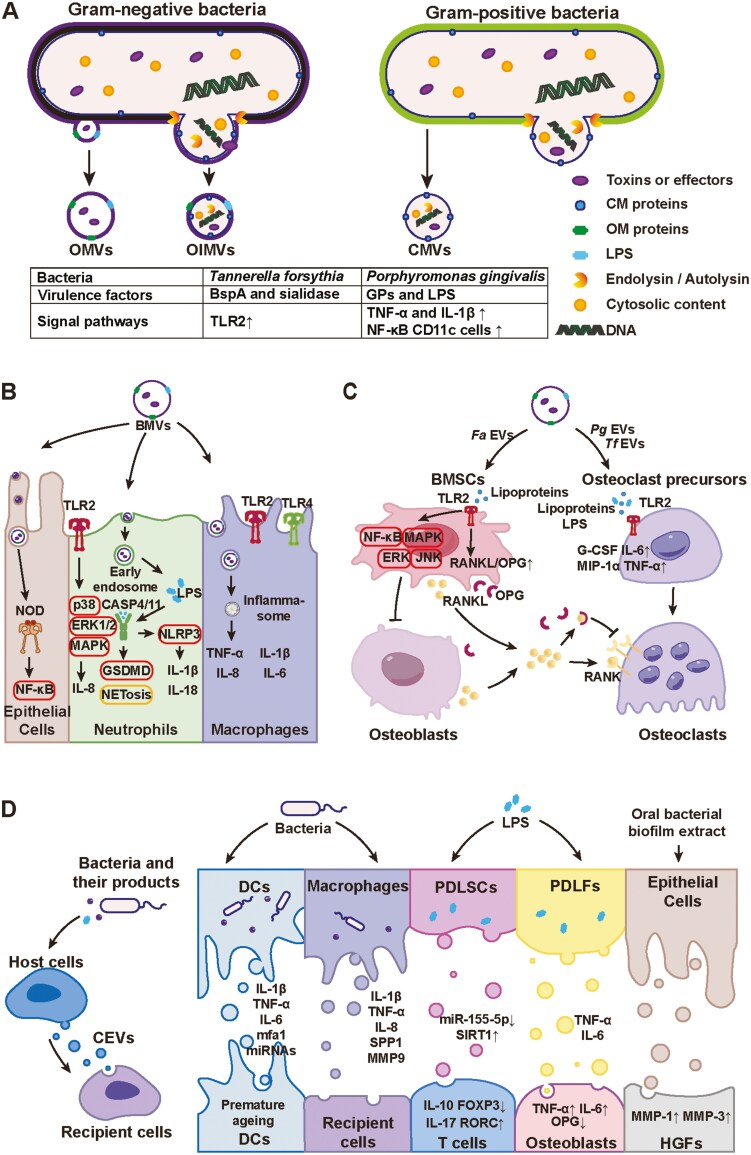
Pathogenic roles of extracellular vesicles in the pathogenesis of periodontal disease. (A) The biogenesis of bacterial-derived extracellular vesicles (BEVs) containing various types of virulence factors. Outer membrane vesicles (OMVs) and outer-inner membrane vesicles (OIMVs) are generated by gram-negative bacteria, whereas cytoplasmic membrane vesicles (CMVs) originate from gram-positive bacteria. All of these BEVs are capable of transporting virulence factors. For example, OMVs from *T. forsythia* are known to carry BspA and sialidase, which induce inflammation by activating Toll-like receptor 2 (TLR2). Additionally, *P. gingivalis*-derived periplasmic vesicles (*Pg* EVs) contain virulence factors such as gingipain (GP) and lipopolysaccharide (LPS), which increase the expression of tumor necrosis factor-alpha (TNF-α) and interleukin-13 (IL-13) and increase the number of nuclear factor kappa-light-chain-enhancer of activated B cells (NF-κB)-positive CD11c cells within periodontal tissues. (B) Amplification of the immune response mediated by BEVs released from periodontal pathogens. BEVs can bind to host cells (epithelial cells, neutrophils, and macrophages) through interactions with pattern recognition receptors (PRRs), such as TLR2, TLR4, NOD1, and NOD2, thereby initiating the activation of downstream proinflammatory signaling pathways. (C) BEVs induce bone resorption by activating TLR2 in bone mesenchymal stem cells (BMSCs) and osteoclasts, thereby playing dual regulatory roles in bone metabolism. BEVs inhibit osteoblast differentiation by modulating the RANKL-RANK-OPG signaling axis while promoting osteoclast differentiation by upregulating factors for osteoclast precursor cells. (D) Cellular extracellular vesicles (CEVs) originating from host cells infected by bacteria or their products exacerbate the progression of periodontitis. Host cells exposed to bacteria, BMVs, or LPS release CEVs enriched with proinflammatory cytokines and mediators, including TNF-α and interleukin-6 (IL-6). These CEVs further act on recipient cells to intensify inflammation and promote tissue destruction. Abbreviations: CM, cytoplasmic membrane; ERK, extracellular signal-regulated kinase; *Fa*, *Fusobacterium nucleatum*; FOXP3, Forkhead Box P3; G-CSF, granulocyte colony-stimulating facto; GSDMD, Gasdermin D; IL-10, interleukin-10; IL-17, interleukin-17; IL-1ra, interleukin-1ra; JNK, c-Jun N-terminal kinase; MAPK, mitogen-activated protein kinase; MIP-1α, macrophage inflammation protein-1α; MMP1, matrix metalloproteinase-1; MMP3, matrix metalloproteinase-3; MMP9, matrix metalloproteinase-9; NLRP3, NOD-like Receptor Family Pyrin Domain Containing 3. OM, outer membrane; OPG, osteoprotegerin; RANK, receptor activator of nuclear factorκB; RANKL, the receptor activator of nuclear factorκB ligand; RORC, Retinoic Acid Receptor-related Orphan Receptor C; SIRT1, Sirtuin-1; SPP1, Secreted Phosphoprotein 1; *Tf*, *Treponema denticola*

Bacteria can produce BEVs by either shedding the outer membrane or explosive cytolysis. The BEVs produced through blebbing of the outer membrane can be categorized into the following three types: outer membrane vesicles (OMVs), outer-inner membrane vesicles (OIMVs), and cytoplasmic membrane vesicles (CMVs)^21^ (Figure 1A). Outer-inner membrane vesicles arise from blebbing of the outer membrane due to an imbalance in peptidoglycan biosynthesis, denatured protein accumulation, or the insertion of hydrophobic molecules into the outer membrane. Intact peptidoglycans and the inner membrane can prevent the entry of cytoplasmic constituents into OMVs; therefore, the cargos of OMVs are devoid of cytoplasmic components but rich in periplasmic and outer membrane proteins.^22^ When the bacterial peptidoglycan layer is weakened by autolysin, the cytoplasmic contents, inner membranes, and cytoplasmic contents can be enveloped by OIMVs. CMVs may form as a result of stress-induced autolysis by gram-positive bacteria or peptidoglycan hydrolysis caused by exogenous endolysin or antibiotics that inhibit peptidoglycan biosynthesis.^21^ Although the biogenesis mechanisms of OMVs, OIMVs, and CMVs are substantially different, distinguishing these vesicles is difficult. In addition, pure OMVs, OIMVs, and CMVs cannot be obtained via current isolation methods. Therefore, OMVs, OIMVs, and CMVs are collectively referred to as BEVs in this study.^17^

#### Transmission and enrichment of virulence factors

BEVs can carry and transport many toxins or virulence factors, actively participate in periodontitis-related tissue damage and deliver many effectors to host tissues that are not colonized by parental bacteria.^17^ Membrane-anchored toxins, including lipopolysaccharides (LPSs) and gingipains, along with bacterial surface adhesion factors, can be integrated into the membranes of OMVs and OIMVs and play key roles in the BEV-mediated pathogenic effects associated with periodontitis. Additionally, OIMVs or CMVs contain bacterial nucleic acids (eg, RNA and DNA) and cytoplasmic proteins and enzymes (eg, peptidyl arginine deiminase), which serve as virulence factors derived from intracellular components. The interplay between virulence factors and BEVs is critically important in the development of periodontal diseases. For example, the OMVs of *Tannerella forsythia* carry harmful substances such as BspA and sialidase, causing inflammation by activating TLR2 in macrophages.^23^ EVs derived from *Porphyromonas gingivalis* contain virulence factors, such as gingipains and LPS, which induce inflammation and increase the levels of TNF-α and interleukin-1β (IL-1β) expression in gingival tissues (Figure 1A). Furthermore, the virulence factors of BEVs are implicated in Alzheimer’s disease, colitis, and dysbiosis of the intestinal microbiota.^24-26^*Aggregatibacter actinomycetemcomitans* is implicated in aggressive forms of periodontitis, and OMVs derived from *A. actinomycetemcomitans* can deliver biologically active virulence factors (CDT, OmpA) into human gingival fibroblasts (HGFs) and can activate nucleotide-binding oligomerization domain-containing protein 1 (NOD1) and 2 (NOD2), thus triggering an excessive innate immune response^27-29^ (Figure 1A).

#### Amplification of immune responses

Upon bacterial infection of the host organism, both the bacteria and their released vesicles can activate the immune response. BEVs can directly activate a broad range of innate immune PRRs, including surface Toll-like receptors (TLRs) and cytosolic NOD1 and NOD2, and then activate the nuclear factor kappa-light-chain-enhancer of activated B cells (NF-κB) and inflammasomes, resulting in the production of proinflammatory cytokines, inflammation, and programmed cell death across various cell types, such as epithelial cells, neutrophils, monocytes, and macrophages^21,30^ (Figure 1B).

Neutrophils are one of the most important immune cells that defend against bacterial invasion. Gasdermin D (GSDMD), a pore-forming protein, has emerged as a key downstream effector of pyroptosis, a form of cell death triggered by intracellular LPS. After the endocytosis of OMVs by neutrophils, LPS on the surface of OMVs can induce NETosis through GSDMD.^15^ LPS binds to and activates caspase-4/11 within the noncanonical inflammasome complex, leading to the cleavage of GSDMD, which subsequently causes perforation of the nuclear and plasma membranes. This altered nuclear permeability allows caspase-11 to enter chromatin, leading to histone cleavage, inactivation and DNA amplification. Disruption of the plasma membrane enables neutrophils to expel their uncompressed chromatin. In addition, this process can trigger the activation of the NLRP3 inflammasome and the secretion of IL-1β and interleukin-18 (IL-18). During this process, caspase-4/11-driven NETosis prevents cytoplasmic infection, whereas the expulsion of neutrophil extracellular traps (NETs) results in the dissemination of bacteria within the host.^15,31^ Nevertheless, the OMVs of bacteria also facilitate bacterial evasion of the immune system. For example, *P. gingivalis* produces gingipains, which are cysteine proteinases that differentially affect interleukin-8 (IL-8) depending on their form. In their soluble form, gingipains first convert IL-8 into a more potent form and then gradually degrade it. However, when attached to OMVs, these molecules instantly degrade IL-8. This difference in activity can result in localized proinflammatory and anti-inflammatory responses around bacterial plaques, which may explain why the infection persists despite high levels of neutrophils at sites of periodontitis.^32^ In addition to IL-8, gingipains ensure bacterial survival by degrading secretory granule components with antibacterial activity produced by neutrophils, particularly the antimicrobial peptides leucine‒leucine-37 (LL-37) and myeloperoxidase (MPO).^33^

In addition to neutrophils, macrophages can engulf vesicles, with OMVs from *P. gingivalis*, *Treponema denticola*, and *Fusobacterium nucleatum* being engulfed by monocytes and macrophages in a dose-dependent manner. These OMVs also activate NF-κB and augment the release of proinflammatory cytokines such as tumor necrosis factor alpha (TNFα), IL-8, and IL-1β.^34^ OMVs from *F. nucleatum* polarize macrophages toward the M1 phenotype, thereby exacerbating the development of periodontitis.^35^

#### Bone resorption

Bone homeostasis is maintained by the coordinated action of two different types of cells: osteoblasts and osteoclasts. Osteoblasts, which originate from the mesenchymal lineage, play a crucial role in bone formation and facilitate mineralization by secreting unmineralized bone matrix and noncollagenous proteins, whereas osteoclasts, which originate from the hematopoietic lineage, are essential for bone resorption because they release hydrogen ions and lytic enzymes.^36,37^ In the context of periodontitis, under the dual pressures of microbial dysbiosis and an excessive immune response, the coordinated activity of osteoblasts and osteoclasts is disrupted, resulting in inhibited bone formation and increased bone resorption.^38^

The critical regulatory axis for bone homeostasis, namely, the receptor activator of nuclear factor κB ligand (RANKL)-receptor activator of nuclear factor κB (RANK)-osteoprotegerin (OPG) axis, is essential for periodontitis-related bone resorption. RANKL is synthesized by multiple cellular lineages, with osteoblasts and bone marrow stromal cells (BMSCs) serving as principal sources within osseous tissue.^39^ Throughout the lifespan of multinucleated osteoclasts, RANK expression persists on cells in the osteoclast lineage until terminal differentiation into mature entities. The interaction of RANKL and RANK not only induces osteoclastogenesis but also promotes the survival and activation of these cells, thereby facilitating osteoclastic resorption. Additionally, osteoblasts secrete OPG, which functions as a soluble decoy receptor for RANKL, competitively inhibiting the binding of RANKL to its cognate receptor RANK. This regulatory mechanism mitigates osteoclast formation while promoting the apoptosis of previously differentiated and activated osteoclasts, ultimately decreasing the extent of bone resorption. Therefore, the RANKL/OPG ratio is a crucial factor influencing the rate and extent of osteoclast-mediated bone resorption.^40^

BEVs derived from *Filifactor alocis* inhibit osteogenic differentiation by activating TLR2 and subsequently triggering the MAPK and NF-κB signaling pathways. During this process, extracellular signal-regulated kinase (ERK) and c-Jun N-terminal kinase (JNK) serve as the principal downstream effectors of TLR2 (Figure 1C). Additionally, BEVs augment the RANKL/OPG ratio in a TLR2-dependent manner, thereby promoting osteoclast differentiation and subsequently increasing bone resorption.^14,39^ In contrast, the BEVs of *F. nucleatum* increased the number of osteoclasts and exacerbated alveolar bone loss in a mouse model of periodontitis. BEVs from *P. gingivalis* and *T. forsythia* promote osteoclast differentiation of osteoclast precursors and increase the expression of osteoclastogenic cytokines, such as granulocyte colony-stimulating factor (G-CSF), interleukin-1ra (IL-1ra), interleukin-6 (IL-6), IFN-γ-inducible protein 10 (IP-10), macrophage inflammation protein-1α (MIP-1α), macrophage inflammation protein-1β (MIP-1β), and macrophage inflammation protein-2 (MIP-2), which are regulated upon activation and normally expressed by T cells (RANTES) and TNF-α (Figure 1C). Additionally, the osteoclastogenic effect induced by BEVs can be attenuated by lipoprotein lipase and polymyxin B (a drug that inhibits LPS). These findings indicate that BEVs from *P. gingivalis* and *T. forsythia* promote osteoclastogenesis by activating TLR2 via lipoprotein/LPS interactions^41^ (Figure 1C).

### Pathogenic roles of CEVs in periodontal homeostasis

The influence of bacteria on periodontal homeostasis extends beyond the bacteria themselves and their released BEVs. Bacteria can also exploit host cells to secrete CEVs containing proinflammatory cytokines and mediators, thereby amplifying the inflammatory response. For example, the expression of age-related microRNAs (miRNAs), IL-1β, IL-6, TNF-α, and the *P. gingivalis* fimbrial adhesin protein Mfa1 in EVs increases significantly when dendritic cells (DCs) are exposed to *P. gingivalis*, which elicits premature senescence in neighboring DCs through paracrine signaling^42,43^ (Figure 1D). In the context of *T. forsythia* infection, macrophage-derived CEVs are rich in proinflammatory cytokines and inflammatory mediators, such as TNF, IL-1β, HLA class I histocompatibility antigen B-7 alpha chain (HLA-B) and matrix metalloproteinase-9 (MMP9), which contribute to an exaggerated inflammatory response during the progression of periodontitis.^23^ Under normal conditions, periodontal ligament stem cells (PDLSCs) can mitigate the inflammatory microenvironment via the T helper 17 (Th17) cell/regulatory T-cell (Treg)/miR-155-5p/Sirtuin-1 (SIRT1) regulatory network. However, EVs derived from LPS-stimulated PDLSCs presented decreased expression of miR-155-5p and elevated levels of SIRT1, resulting in an imbalance between Th17 cells and Tregs.^44^ Similarly, the upregulation of TNF-α and IL-6 in EVs derived from LPS-stimulated periodontal ligament fibroblasts (PDLFs) increased significantly, which inhibited alkaline phosphatase, collagen I, Runt-related transcription factor 2, and osteoprotegerin expression along with ALP activity in MG-63 osteoblasts, leading to decreased bone formation^45^ (Figure 1D). Moreover, EVs released from biofilm-stimulated gingival epithelial cells significantly upregulated the expression of matrix metalloproteinase-1 (MMP-1) and 3 (MMP-3) in HGFs, which promoted a tissue destruction phenotype and reduced the production of the extracellular matrix (ECM), leading to increased tissue degradation^46^ (Figure 1D). These findings indicate that CEVs also play key pathogenic roles in the imbalance of periodontal homeostasis.

## Anti-inflammatory effects of MSC-EVs on immune cells

**Figure 2.**
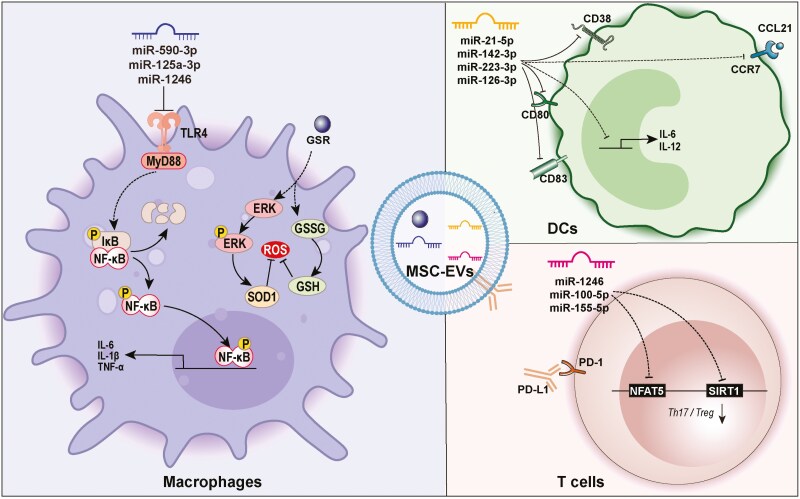
Anti-inflammatory effects of MSC-EVs on immune cells. MSC-EVs can transfer diverse cargoes (such as miRNAs and proteins) to different immune cells (including macrophages, T cells, and dendritic cells). These cargoes can activate various signaling pathways to exert anti-inflammatory effects on immune cells. Abbreviations: CCL21, C-C motif chemokine ligand 21; CCR7, chemokine receptor 7; ERK, extracellular signal-regulated kinase; GSH, glutathione; GSR, glutathione reductase; GSSG, glutathione disulfide; IL-12, interleukin-12; IL-1β, interleukin-1β; IL-6, interleukin-6; IκB, Inhibitor of κB; MyD88, Myeloid Differentiation Primary Response 88; NFAT5, Nuclear Factor of Activated T-cells 5; NF-κB, the nuclear factor kappa-light-chain-enhancer of activated B cells; PD-1, programmed cell death protein 1; PD-L1, programmed death ligand 1; ROS, reactive oxygen species; SIRT1, Sirtuin 1 SOD1, superoxide dismutase 1; TLR4, Toll-like receptor 4; TNF-α, tumor necrosis factor-alpha.

A balanced host immune response is the critical driver of periodontal homeostasis and health.^47^ MSC-EVs exert a profound and far-reaching effect on the behaviors of immune cells. These vesicles play pivotal roles in modulating macrophage polarization, steering macrophages from the M1 proinflammatory phenotype to the M2 anti-inflammatory phenotype. This transition plays a vital role in managing inflammatory responses and enhancing tissue repair. MSC-EVs also strongly impact T-cell regulation, notably affecting the differentiation of CD4 + T cells into Tregs. Additionally, MSC-EVs are pivotal in reducing the secretion of proinflammatory cytokines by DCs, decreasing their migratory capacity, and bolstering their anti-inflammatory functions. The intricate interactions between immune cells further amplify these regulatory effects; for example, MSC-EVs can indirectly influence T-cell differentiation by fostering anti-inflammatory responses in both macrophages and DCs. As a result, the modulation of immune cell behavior by MSC-EVs shows promise for therapeutic applications in managing inflammation and promoting tissue regeneration (Figure 2).

### Macrophages

Although there is a continual spectrum of macrophage phenotypes, macrophages can be classically divided into two phenotypes: the M1 proinflammatory phenotype and the M2 anti-inflammatory phenotype. These phenotypes are typically associated with wound healing, and the macrophage transition from the M1 phenotype to the M2 phenotype effectively alleviates inflammatory responses and promotes periodontal tissue regeneration.^48^ After systematic injection, MSC-EVs are engulfed mainly by macrophages, and the bioactive cargos loaded with MSC-EVs can repolarize macrophages from the M1 to the M2 phenotype through multiple signaling pathways. For example, EVs derived from dental pulp stem cells (DPSC-EVs) directly target inhibitor of nuclear factor kappa B kinase subunit beta (IKBKB) to inhibit the NF-κB and TLR signaling pathways, which inhibits the proinflammatory responses of macrophages and induces macrophage polarization toward the M2 phenotype. Moreover, these vesicles promote the release of bone morphogenetic protein 2 (BMP2) from macrophages to induce odontogenesis in dental pulp stem cells (DPSCs).^49^ Similarly, miR-1246 and miR-125a-3p in DPSC-EVs promoted the polarization of macrophages toward an anti-inflammatory phenotype in periodontitis models.^50,51^ Moreover, MSC-EVs can inhibit the pyroptosis of macrophages to inhibit inflammation, and EVs produced by PDLSCs facilitate the delivery of miR-590-3p to macrophages, resulting in the inhibition of TLR4 transcription and a subsequent reduction in macrophage pyroptosis, thereby mitigating the inflammatory damage caused by periodontitis.

In addition, pretreatment with MSCs can promote the anti-inflammatory effects of MSC-EVs in modulating macrophage function. For example, EVs derived from LPS-preconditioned dental follicle cells (DFC-EVs) inhibited reactive oxygen species (ROS) formation and promoted macrophage polarization toward the M2 phenotype through the activation of ROS/ERK signaling.^52^ Moreover, TNF-α pretreatment promoted the exosomal expression of CD73 to induce M2 macrophage polarization, thereby facilitating inflammation and periodontal bone preservation.^53^ Similarly, TNF-α-pretreated MSC-EVs decreased the levels of proinflammatory markers including IL-1β and inducible nitric oxide synthase (iNOS), while increasing the expression of reparatory markers such as Arg1 and CD206. This effect is potentially mediated by miRNAs within the EVs, promoting an immunomodulatory role.^54^ On the basis of these findings, Deng et al^55^ achieved sustained modulation of macrophage polarization by immobilizing MSC-EVs in hydrogels through biotin-avidin systems, reducing the expression of M1 polarization-associated proteins like iNOS and chemokine receptor 7 (CCR7), and increasing the levels of the M2 polarization-associated protein CD206, thereby promoting periodontal tissue repair. These studies deepen our understanding of the mechanisms by which EVs influence macrophage polarization and highlight their potential in modulating immune responses and promoting tissue repair.

### T cells

T cells are among the most abundant cells in healthy and diseased periodontal tissues.^56^ In patients with periodontitis, the balance between Th17 cells and Tregs in the peripheral blood is disrupted, resulting in increased inflammation and alleviated tissue damage.^44^ MSC-EVs have considerable potential in the modulation of immune responses, particularly in the induction of Tregs and reduction of T-cell proliferation as well as related interferon gamma (IFN-γ) release.^57^ MSC-EVs directly influence T-cell activation and differentiation by expressing specific proteins at elevated levels. Notably, Wharton’s jelly-derived MSCs, when stimulated with IFN‐γ, increased the secretion of EVs carrying programmed death-ligand 1 (PD-L1). PD-L1 expressed by MSC-EVs interacts with programmed death receptor 1 (PD1) on T cells, thereby influencing T-cell receptor (TCR)-mediated activation and contributing to the mitigation of acute graft-versus-host disease (aGvHD).^58^ Furthermore, MSC-EVs express galectin-1 and transforming growth factor beta (TGF-β), which have been shown to induce the apoptosis of activated T cells and encourage the generation of Tregs.^59^ In contrast, MSC-EVs can indirectly modulate T-cell differentiation by modulating the cellular behavior of macrophages. In vitro studies demonstrated that EVs from human umbilical cord MSCs (HUCMSCs) could suppress the proliferation of CD4+ T cells and enhance the generation of Tregs by educating macrophages toward the M2-like phenotype. From a mechanistic perspective, miR-100-5p enriched in EVs promoted M2 macrophage polarization and diminished their ability to suppress T cells and promote Treg expansion. Knocking down miR-100-5p in MSC-sEVs partially inhibited the ability of HUCMSC-sEVs to promote M2 macrophage and Treg expansion.^60^

### Other immune cells

Following inflammatory stimulation, MSC-EVs are also internalized more by innate immune effector cells (IECs), possibly because of their heightened expression of intercellular adhesion molecule-1 (ICAM-1), which allows MSC-EVs to bind to target cells.^61^ Once internalized by IECs, MSC-EVs exert immunomodulatory effects by regulating the expression of inflammatory factors. For example, treatment with MSC-EVs hindered antigen uptake by immature DCs and suppressed their maturation, leading to reduced levels of the maturation and activation markers CD83, CD38, and CD80. Moreover, this treatment results in decreased levels of the proinflammatory cytokines IL-6 and IL-12p70 while simultaneously increasing the production of the anti-inflammatory cytokine TGF-β. Additionally, MSC-EV-incubated DCs presented reduced CCR7 expression following LPS stimulation and a notably diminished ability to migrate toward the CCR7 ligand C-C motif chemokine ligand 21 (CCL21). The underlying mechanism involves the ability of miR-21-5p within MSC-EVs to degrade CCR7, and miR-21-5p mimic transfection significantly decreases the ability of DCs to migrate toward CCL21.^62^ Another study indicated that MSC-EVs promoted the content and secretion of IL-6 and interleukin-10 (IL-10) by DCs, resulting in a decreased population of Th17 cells and an increased number of Treg cells. Furthermore, MSC-EV-treated T cells exhibited elevated production of anti-inflammatory cytokines, including IL-10, TGF-β, and IL-6.^63^

## Repair-promoting functions of MSC-EVs in periodontal tissue regeneration

**Figure 3.**
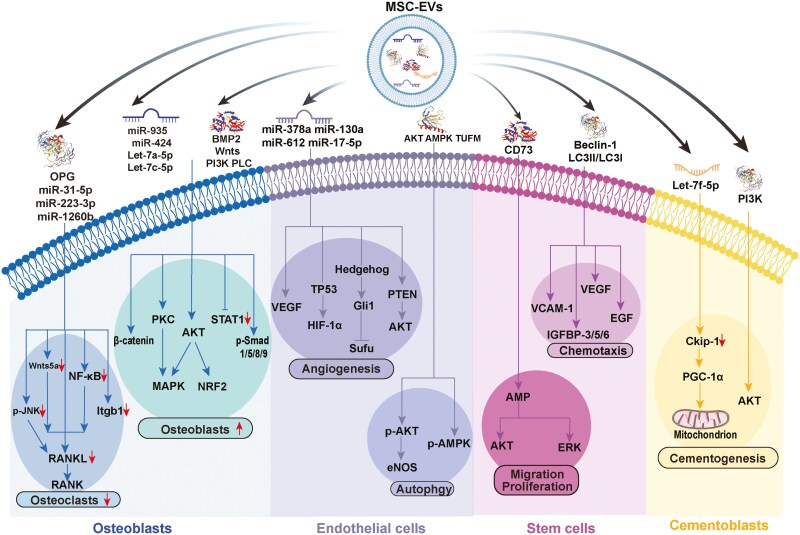
The therapeutic effects of MSC-EVs on periodontitis. MSC-EVs deliver multiple cargoes (eg, miRNAs, proteins, etc.) to different recipient cells, including osteoblasts, cementoblasts, MSCs, and ECs. Multiple underlying mechanisms are involved in MSC-EV-mediated osteogenesis, angiogenesis, cell migration/proliferation, and cementogenesis. These signaling pathways regulate biological processes in recipient cells to exert therapeutic effects on periodontitis. Abbreviations: AKT, protein kinase B; AMP, adenosine monophosphate; AMPK, AMP-activated protein kinase; BMP2, bone morphogenetic protein 2; Ckip-1, casein kinase 2 interacting protein-1 EGF, epidermal growth factor; ERK, extracellular signal-regulated kinase; HIF-1α, hypoxia-inducible factor 1-alpha; IGFBP-3/5/6, insulin-like growth factor binding protein-3/5/6; MAPK, mitogen-activated protein kinase; NF-κB, the nuclear factor kappa-light-chain-enhancer of activated B cells; OPG, osteoprotegerin; p-AKT, the phosphorylation of AKT; p-AMPK, the phosphorylation of AMPK; PI3K, phosphoinositide 3-kinase; p-JNK, the phosphorylation of c-Jun N-terminal kinase; PKC, protein kinase C; PLC, phospholipase C; p-Smad1/5/8/9, the phosphorylation of Smad1/5/8/9; RANK, receptor activator of nuclear factor κB; RANKL, the receptor activator of nuclear factor κ ligand; STAT1, signal transducer and activator of transcription 1; TUFM, Tu translation elongation factor; VCAM-1, vascular cell adhesion molecule-1; VEGF, vascular endothelial growth factor; Wnts, wingless protein.

Many studies have emphasized that MSC derivatives, such as EVs, Exos, matrix vesicles (MVs), and apoptotic bodies (ABs), can be used as alternatives to MSCs to promote periodontal tissue regeneration because of their strong repair-promoting functions.^48,64-66^ MSC-EVs can exert multiple bioactivities, including pro-osteogenic/cementogenic, proangiogenic, and pro-stem cell migratory, proliferative and multilineage differentiation effects, to facilitate the repair of complex periodontal tissues.^67^ This section summarizes the repair-promoting functions of EVs in different periodontal tissues and the underlying mechanisms (Figure 3 and Table 1).

**Table 1.** Summary of EVs used in periodontal tissue regeneration.

Cell source	EVs type	Isolation protocol	Cargoes	Effects	Mechanisms	Ref.
BMSC	EVs	Gradient ultracentrifugation	Bgn	Promote osteoblast proliferation, migration, differentiation, and mineralization	PI3K/AKT	^81^
M2 Macrophage	Exos	Differential centrifugation	Let-7f-5p	Promote cementoblast mineralization	Downregulation of Ckip-1 by Let-7f-5p activates mitochondrial biogenesis	^118^
MSC	EVs	Differential centrifugation	miR-612	Promote ECs migration, proliferation and angiogenesis	miR-612/TP53/HIF-1α	^121^
BMSC	EVs	Polymer precipitation, centrifugation techniques	BMP2	Induce osteoinduction and bone regeneration	Phosphorylation of Smad 1/5/8	^95^
DPSC	Abs	Differential centrifugation	TUFM, TFEB	Promote ECs proliferation and angiogenesis	The TFEB-induced autophagy-lysosome pathway	^125^
BMSC	EVs	Polymer precipitation, centrifugation techniques	OPG	Promote PDLSCs proliferation, migration and differentiation; Inhibit osteoclast activity; promote M2 macrophage polarization	OPG/RANKL/RANK	^84^
PDLSC	Exos	Differential centrifugation	miR-31-5p	Inhibit osteoclast formation and reduce bone destruction	miR-31-5p/eNOS	^87^
GMSC	Exos	Immunoprecipitation, ultracentrifugation	miR-1260b/CD73	Promote M2 macrophage polarization and enhance wound healing; Promote osteogenesis and inhibit bone loss	Wnt5a/JNK/RANKL	^53^
DPSC	EVs	Ultracentrifugation	miR-1246	Promote osteogenesis	Upregulation of miR-1246	^107^
SCAP	EVs	Ultracentrifugation, differential centrifugation	miR-935	Promote osteoblast differentiation of PDLSCs; inhibit inflammation and bone resorption	Upregulation of miR-935	^109^
SHED	Exos	Ultracentrifugation, differential centrifugation	β-catenin, Wnt3a, BMP2	Promote osteoblast differentiation of PDLSCs	Wnt/β-catenin; BMP/Smad (the phosphorylation of Smad 1/5/8)	^98^
BMSC	EVs	Polymer precipitation,ultrafiltration	miR-424	Promote bone formation	Phosphorylation of Smad 1/5/8	^96^
BMSC	Exos	Ultracentrifugation	DPT-C, miR-26a	Promote osteogenesis, proliferation and migration of BMSCs	mTOR pathway/Wnt/autophagy	^91^
BMSC	Abs	Differential centrifugation	miR-223-3P	Inhibit osteoclast activity and alleviate bone destruction	Target Itgb1	^64^
BMSC, ASC	Exos	Gradient ultracentrifugation	let-7a-5p, let-7c-5p, miR-328a-5p, miR-31a-5p	Promote osteoblast differentiation of BMSCs	Targeted Acvr2b/Acvr1 and regulate the balance of Bmpr2/Acvr2b for Bmpr-elicited Smad1/5/9 phosphorylation	^97^
BMSC	Exos	Ultracentrifugation	miR-130a	Promote ECs migration	PTEN/AKT; Wnt/β-catenin NF-κB/AKT	^124^
SHED	EVs	Differential centrifugation	Chemotactic Factors (TGF-β1, IGF-1, FGF-2, PDGF-BB)	Promote migration of BMSCs	Chemotactic effect	^129^
DPSC	EVs	Differential centrifugation	miR-758-5p	Promote osteoblast differentiation of PDLSCs	miR-758-5p/LMBR1/BMP2/4	^94^
PDLSC	Exos	Ultracentrifugation	miR-17-5p	Increase VEGFA and promote tube formation of ECs	Downregulation of miR-17-5p	^122^
DPSC	EVs	Ultracentrifugation	miR-378a	Promote ECs migration, proliferation, tube formation and angiogenesis	miR-378a/Sufu/Hedgehog/Gli1	^123^

**Abbreviations:** ABs, apoptotic bodies; AKT, protein kinase B; ASC(ADSC), adipose-derived stem cells; BMP2, bone morphogenetic protein 2; BMSC, bone mesenchymal stem cells; DPSC, dental pulp stem cells; ECs, endothelial cells; EVs, extracellular vesicles; Exos, exosomes; GMSC, gingival mesenchymal stem cells; HIF-1α, hypoxia-inducible factor 1-alpha; JNK, c-Jun N-terminal kinaseMSC, mesenchymal stem cells; OPG, osteoprotegerin; PDLSC, periodontal ligament stem cells; PI3K, phosphatidylinositide 3-kinase; RANK, receptor activator of nuclear factor κ; RANKL, receptor activator of nuclear factor κB ligand; SCAP, stem cells from the apical papilla; SHED, stem cells from human exfoliated deciduous teeth; TUFM, Tu translation elongation factor; VEGFA, vascular endothelial growth factor A; Wnt3a, wingless protein 3a.

### Pro-osteogenic/cementogenic effects

Periodontitis is a long-lasting inflammatory disease characterized by the permanent loss of tissues that support the structures of teeth. If left untreated, this condition can lead to tooth loss^68^; thus, restoring the lost supporting tissues is the primary objective of periodontitis treatment. Achieving complete periodontal tissue regeneration is a challenging objective, which necessitates addressing the complex anatomical structure of periodontal tissue^69^ and managing the uncontrolled inflammatory microenvironment.^70^ Notably, the delivery of bioactive cargoes, which are involved in the mediation of pro-osteogenic and cementogenic effects, to recipient cells is emerging as a promising cell-free therapeutic strategy to address this challenge. MSC-EVs have been shown to be effective in various animal models of periodontitis.^54,71-78^

Prior to the introduction of EVs into the clinical setting, a comprehensive understanding of the underlying mechanisms, with different pathways playing pivotal roles in the osteogenesis of PDLSCs, is essential. The phosphatidylinositide 3-kinase/protein kinase B (PI3K/AKT) pathway is known to mediate bone metabolism,^79^ and almost all types of EVs derived from different stem cells (eg, MSCs, PDLSCs, BMSCs and HUCMSCs) can promote the bone-generating differentiation of PDLSCs by triggering this process.^80-82^ For example, Zhai et al.^80^ demonstrated that MSC-EVs could enter MSCs via caveolin-1 and promote osteogenesis by stimulating the PI3K/AKT/MAPK signaling pathway. Similarly, adipose-derived stem cell (ADSC)-derived EVs may promote the osteogenesis of MSCs by eliminating cellular ROS through activation of the PI3K/AKT/NRF2 pathway and reducing mitochondrial changes/DNA damage.^83^ In addition to the PI3K/AKT pathway, the RANKL-RANK-OPG axis is another critical regulatory axis for bone homeostasis, as noted above. Numerous studies have demonstrated that EVs extracted from dental follicle stem cells (DFSCs), BMSCs, gingival mesenchymal stem cells (GMSCs) or cementocytes can inhibit osteoclast activity and promote osteoblast differentiation by decreasing the ratio of RANKL/OPG expression^52,84^ or inhibiting related key signals such as NF-κB,^85,86^ wingless protein (Wnt) 5a or the phosphorylation of JNK.^53^ Some miRNAs, such as those in the miR-223-3p/Itgb1 axis,^64^ the miR-31-5p/eNOS signaling pathway^87^ and the miR-503-3p/HPSE axis,^88^ are also involved in this process. Furthermore, miRNA-involved axes play crucial roles in promoting osteogenesis and inhibiting osteoclastogenesis; for example, BMSC-Exos delivered high levels of miR-935 to stimulate osteoblast proliferation and differentiation by inhibiting signal transducer and activator of transcription 1 (STAT1),^68^ and PDLSC-Exos promoted osteogenic differentiation by inhibiting TNF via Exos carrying miR-181d-5p.^89^ Among these osteogenesis-related pathways, Wnt signaling represents a classic pathway involved in maintaining periodontal tissue homeostasis^90^; however, the role of this pathway remains controversial. Most relevant experiments have demonstrated that stimulation of the Wnt/β-catenin pathway is a prerequisite for the pro-osteogenic effects of EVs from BMSCs and PDLSCs.^91,92^ However, a study conducted by Lei et al.^93^ revealed that excessive activation of Wnt signaling in PDLSCs impaired osteogenesis during periodontitis. These contradictory results may be attributed to the different origins of MSCs and varying culture conditions (with or without inflammatory factors),^93^ which necessitates further investigation in the future. Moreover, the bone morphogenetic protein (BMP)/Smad pathway is equally essential for osteogenesis, and EVs from the inflamed microenvironment increase the osteoblast differentiation of PDLSCs partly by inhibiting LMBR1-targeting miR-758-5p via BMP signaling.^94^ In addition, because of the targeting role of multicomponent EV miRNAs and proteins (eg, BMP2, let-7a-5p and miR-424), EVs derived from various types of MSCs [eg, BMSCs,^95,96^ ADSCs,^97^ and stem cells from human exfoliated deciduous teeth (SHEDs)^98]^ can activate the phosphorylation of Smad 1/5/8/9 to promote bone regeneration, indicating that EVs share a common underlying mechanism of EV-induced osteogenesis. In addition, research progress on other mechanisms offers promising avenues for the development of bone regeneration in periodontitis, including the activation of the phospholipase C/protein kinase C/ (PLC/PKC/MAPK) pathway,^69^ the p38 MAPK signaling pathway,^99^ the adenosine monophosphate-activated protein kinase (AMPK) pathway^100^ and the AKT-mediating systems.^101-103^ In summary, MSC-derived EVs can activate various osteogenesis-related pathways, resulting in accelerated bone regeneration, which is facilitated by the release of multiple bioactive cargoes.

In addition, multiple mechanisms, including autophagy^104^ and mitochondrial functions,^105^ have been shown to participate in MSC-EV-strengthened osteogenesis. For example, autophagy is a cellular mechanism that allows cells to break down and recycle proteins and organelles to preserve the intracellular balance, is strengthened by hypoxia and then induces rapid release of BMSC-EVs.^104^ The transplantation of BMSC-EVs generated under hypoxic conditions significantly increased the reconstruction of defects in alveolar bone.^104^ Additionally, Dai et al.^105^ demonstrated that Exos obtained from inflammatory PDLSCs can safeguard their mitochondrial functions by increasing the mitochondrial membrane potential and reducing oxidative stress levels, thereby facilitating the osteogenic differentiation of PDLSCs. Furthermore, EVs enriched with specific miRNAs promote osteoclastogenesis by rebalancing the coordinated activities of osteoblasts and osteoclasts, which is achieved through the regulation of relevant gene expression. These altered miRNAs include upregulated miRNAs [eg, hsa-miR-29c-5p,^106^ miR-1246,^107^ miR-21-5p,^108^ miR-935,^109^ and miR-335-5p^110]^ and downregulated miRNAs [eg, hsa-miR-31-3p, hsa-miR-31–3p, hsa-miR-221–3p, hsa-miR-183–5p and hsa-miR-503–5p,^106^ and hsa-miR-125b-5p^111]^, which not only promote the expression of osteoblast-related genes (eg, RUNX, OCN, and BMP) but also inhibit the expression of osteoclast genes, including TRAP, NFATc1, and Cathepsin K. Nevertheless, the quantity of newly formed bone must also be considered. Bone remodeling is an essential aspect of bone repair and involves unhealthy bone resorption^112^ and the inhibition of osteogenesis.^113^ Numerous studies have confirmed that miRNAs and long noncoding RNAs (lncRNAs) are important posttranscriptional regulators that participate in the modulation of bone metabolism, such as the upregulation of miR-28^114^ and lncRNA-MALAT1^110^ or the downregulation of miR-124,^112^ which can increase osteoclast viability; high expression of miR-34c-5p can significantly suppress osteogenesis via the special AT-rich sequence-binding protein 2 (SATB2)/ERK pathway.^113^ Consequently, numerous and various mechanisms are involved in the pro-osteogenic/cementogenic effects of MSC-EVs, but the core of all relevant research is the inhibition of osteoclast generation and differentiation, along with the promotion of osteoblast activity. Therefore, regulating the balance between osteoclasts and osteoblasts is a promising strategy to facilitate the advancement of periodontitis treatment. Additionally, almost all of the above studies were conducted in animal models of periodontitis, but actual human periodontitis is much more complex than this situation is. Therefore, clinical trials on the ability of MSC-EVs to promote osteogenesis/cementogenesis in periodontitis patients need to be conducted in the future.

Although there is a thin layer of mineralized tissue covering the teeth roots, the cementum plays a crucial role in anchoring the teeth within the alveolar socket. This anchoring helps maintain the stability and health of the teeth, which is achieved through the embedded collagen fibers.^115^ Therefore, the regeneration of cementum is widely regarded as the gold standard for periodontal tissue regeneration.^116^ Although cementum is similar to bone in several aspects,^86,90^ differences in its regeneration arise from its unique anatomical position and structure.^65^ Cementoblasts are the principal effector cells responsible for synthesizing cementum, which plays a pivotal role in regulating cementum repair.^117^ Therefore, it is crucial to stimulate cementoblasts to migrate toward root surfaces and promote their cementogenic activity. Exos derived from PDLSCs were shown to increase proliferation, migration, and cementogenesis by triggering the PI3K/AKT signaling pathway.^64^ In addition to Exos derived from MSCs, other sources of Exos, such as M2-like macrophages, promote cementum regeneration. A study conducted by Huang et al.^118^ demonstrated that Exos from Ckip-1-silenced macrophages (sh-Ckip-1-Exos) effectively promoted cementogenesis and rescued *P. gingivalis*-suppressed cementoblast mineralization. The underlying mechanism is that let-7f-5p delivered by sh-Ckip-1-Exos targets Ckip-1, thereby promoting mitochondrial biogenesis in a PGC-1α-dependent manner.^118^ Nevertheless, research on cementum regeneration is lacking. Identification of accessible methods to establish an evaluable cementum-defect model and more characteristic markers to determine the regeneration outcomes of cementum in regeneration systems is urgently needed.

### Proangiogenic effects

Vascularization has been demonstrated to be a prerequisite for bone defect repair.^119^ Mounting evidence has indicated that MSC-EVs can directly or indirectly modulate revascularization to support periodontal tissue regeneration by carrying a panel of miRNAs (eg, increased presence of miR-612 and miR-143-3p and decreased expression levels of miR-15b-5p, miR-16-5p and the let-7-5p family).^120^ For example, MSC-EVs carry miR-612 to promote displacement, proliferation and endothelial cell (EC) tube development by regulating the miR-612/TP53/hypoxia-inducible factor 1-alpha (HIF-1α) axis.^121^ Notably, vascular endothelial growth factor (VEGF) is the most potent agent that participates in the modulation of vascular ECs, with its expression being increased by the downregulation of miR-17-5p in PDLSC-Exos.^122^ Additionally, DPSC-EVs expressing miR-378a have been shown to decrease the expression of Sufu to promote angiogenesis by activating Hedgehog/Gli1 signaling.^123^ Furthermore, Exos derived from BMSCs strongly promoted the migration of ECs, which was attributed to miR-130a and its downstream effects on the PTEN/AKT pathway.^124^ More importantly, Exos from SHEDs augment the pro-regeneration function of ECs by increasing the levels of angiogenic molecules (eg, VEGF-A, KDR, FGF2, and SDF-1) through the regulation of the AMPK signaling pathway,^100^ which is similar to the AKT/eNOS pathway in PDLSC-Exos.^101^ Moreover, Li et al.^125^ demonstrated that ABs from DPSCs promoted EC autophagy by transferring mitochondrial Tu translation elongation factor (TUFM), thereby increasing the angiogenic potential of ECs. Similarly, HUCMSC-Exos^119^ and DPSC-EVs^126^ were shown to facilitate the migration and proliferation of ECs in rat alveolar bone defect models. Overall, angiogenesis has been reported to be associated with osteogenesis, in which the functions of ECs and VEGF play essential roles. Therefore, regulating the activity of ECs and VEGF might be a candidate strategy for periodontitis treatment.

### Pro-stem cell migratory and proliferative effects

In addition to their roles in osteogenesis and angiogenesis, the migration and proliferation of MSCs play equally significant roles in the regeneration of tissue. As in osteogenesis and angiogenesis, various signaling pathways participate in the modulation of MSC proliferation and migration, including the PI3K/AKT pathway,^81^ the Wnt/β-catenin pathway,^92^ the RANKL/RANK/OPG axis^84^ and the MAPK pathway.^99^ For example, MSC-Exos could increase PDLSC movement and multiplication via CD73-induced activation of adenosine receptors on AKT and ERK signaling.^102^ Furthermore, Liu et al^104^ reported that a hypoxic microenvironment promoted MSC homing by increasing the production of proteins related to autophagy (eg, Beclin-1 and LC3II/LC3I), thereby facilitating the secretion of various chemokines [eg, insulin-like growth factor binding protein (IGFBP)-3, IGFBP-6, IGFBP-5, vascular cell adhesion molecule (VCAM)-1, epidermal growth factor (EGF) and VEGF]. EVs derived from various MSCs increase the quantity of specific cytokines, including miRNAs (eg, hsa-miR-29c-5p, hsa-miR-378a-5p, hsa-miR-10b-5p and hsa-miR-9-3p),^106^ nuclear factor I/C (NFIC)^127^ and chemotactic factors (eg, TGF-β1, IGF-1, FGF-2, and PDGF-BB),^126^ which promote the motility of their parental MSCs. In conclusion, MSC-EVs promoted cell migration and proliferation to facilitate complete periodontal tissue regeneration. Hence, the migration of MSCs cannot be ignored when developing therapeutic approaches to promote periodontal regeneration.

However, it remains unclear which types of MSC-EVs exert the most pronounced pro-osteogenic and angiogenic effects among the various types of MSCs, as numerous factors influence their efficacy, including their origin, isolation methods, and culture conditions. Research on the diverse sources of MSC-EVs is limited; however, recent findings suggest that, compared with ADSC-Exos, BMSC-Exos have greater productivity and osteogenic induction.^97^ Compared with GMSCs-EVs, BMSC-EVs do not demonstrate the same advantages.^70^ Additionally, osteoblasts and SHEDs showed superior performance compared with PDLSCs/gingival fibroblasts and DPSCs, respectively.^71,72^ In terms of different isolation methods and culture conditions, Yi et al^69^ compared the biological properties of media microvesicles (MMVs) isolated from culture supernatant and collagenase-released microvesicles (CRMVs) obtained from collagenase-digested cell suspensions. Their results revealed that CRMVs exhibited superior promotion of cell proliferation, migration, mineralization, and osteogenesis.^69^ Additionally, a three-dimensional (3D) culture system was shown to produce greater amounts of Exos than a traditional two-dimensional (2D) system did.^68^ Furthermore, compared with those derived from normoxic MSCs, EVs derived from periodontal DPSCs,^126^ hypoxic BMSCs^55^ and hypoxic MSCs^121^ exhibited increased potential for promoting osteogenesis and angiogenesis. Moreover, Qiu et al^101^ reported that Exos released by neonatal serum-incubated MSCs exhibited a more pronounced angiogenic effect than those released by adult serum-incubated MSCs. In summary, it is imperative to systematically compare the bioactivities of EVs derived from different MSC sources under uniform research conditions to expedite the clinical development of MSC-EVs for tissue regeneration.

## Clinical application of EVs in periodontal tissue regeneration

**Figure 4.**
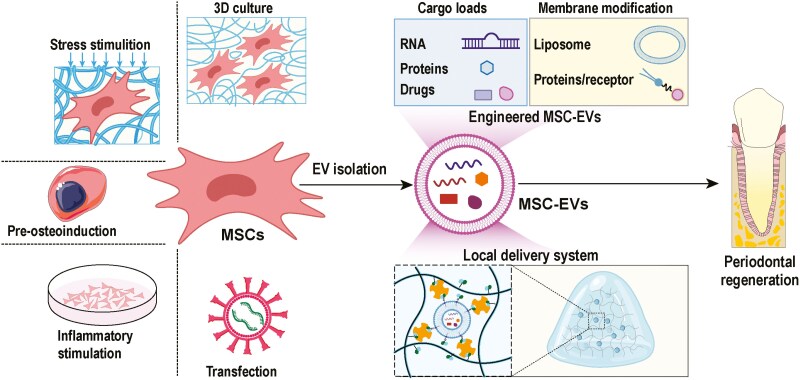
The main strategies for promoting the repair-promoting functions of MSC-EVs paves the way for their clinical application in periodontitis. By preconditioning MSCs, engineering MSC-EVs through cargo loading or membrane modification, and developing local delivery systems for MSC-EVs, researchers can increase the therapeutic efficacy of MSC-EVs, rendering them potentially suitable for the treatment of periodontitis.

The various advantages of MSC-EVs have paved the way for their use in periodontal tissue regeneration. Nevertheless, EV-based therapies are typically limited by their transient half-life and rapid clearance from the site of administration.^70,128^ For increased therapeutic performance of EVs, researchers must augment their bioactive properties or ensure prolonged retention of MSC-EVs at targeted sites. This phenomenon can be achieved through diverse strategies, such as preconditioning of parental MSCs, engineering of MSC-derived EVs, and the development of localized delivery systems (Figure 4). Emerging research has principally explored the potential of preconditioning MSCs to modify key metabolites and proteins in MSC-EVs linked to anti-inflammatory and immunoregulatory functions.^54^ For example, EVs isolated from MSCs preconditioned with TNF-α,^53,54^ gallic acid (GA),^105^ or curcumin^92^ or with the silencing of casein kinase 2 interacting protein-1 (Ckip-1)^118^ or hypoxia^55,121^ showed an increased ability to enhance the displacement, reproduction and osteogenic function of MSCs. In addition, the use of engineered MSC-EVs has demonstrated good potential for increasing EV bioactivity, which allows EVs to constitutively express certain molecules to promote regenerative functions. For example, Huang et al^95^ genetically modified BMSCs by overexpressing BMP2, consequently generating functionally engineered EVs with increased osteoinductive properties; moreover, these researchers modified MSC-EVs to have elevated levels of miR-424, which enhances the BMP2 signaling pathway, leading to improved bone regeneration.^96^ Moreover, polyethylenimine-engineered^77^ and nuclear factor I/C (NFIC)-encapsulated^127^ EVs have better potential for promoting angiogenesis and the migration and proliferation of MSCs, respectively. Furthermore, the targeting, regulated release, and sustained retention of EVs at the desired location have positive effects on periodontal regeneration. Our previously published studies showed that immobilizing MSC-EVs on the side chain of gelatin methacryloyl (GelMA) with the aid of a biotin-avidin system allowed the sustained release of MSC-EXs for up to 28 days. These MSC-EV-functionalized hydrogels significantly increased the ability of MSC-EVs to modulate macrophages and promote tissue repair, leading to the optimized therapeutic performance of MSC-EVs in the regrowth of complex periodontal tissues.^55^ In another study, novel “dual delivery” microparticles were developed to enable the controlled release of EVs in a microenvironment-sensitive manner. This process was achieved by incorporating metalloproteinases to facilitate the secretion of EVs at the impacted position and antibiotics to inhibit bacterial biofilm formation.^70^ This approach strategically modulates the dynamics of EVs release according to the inflammatory condition of the impacted tissue, which can vary across patients, to promote the regeneration of periodontal tissue. More importantly, the complex and dynamic nature of oral tissue healing, characterized by an abundance of inflammatory factors, underscores the need for more effective strategies (such as physical stimulation) to increase the replication and biological activity of MSC-EVs, alongside their pro-osteogenic and anti-inflammatory functions.^109^ In this context, low-intensity pulsed ultrasound (LIPUS)^109^ and cyclic stretch^89^ stimulation were identified as promising methods for further optimization.

In addition to the methods mentioned above, which can increase the quantity and function of EVs, mastering the correct techniques for isolating EVs is crucial. Currently, six main strategies are used to isolate EVs, including ultracentrifugation, immunoaffinity capture, precipitation, ultrafiltration, and size-exclusion chromatography.^130^ Each technique has unique advantages and disadvantages, which influence its applicability in different scenarios. For example, ultracentrifugation is the most highly used method for separating EVs because of its low price and high efficiency,^130^ but it runs a high risk of damaging the structure of EVs.^131^ Other approaches, such as ultrafiltration and precipitation, can avoid disrupting EVs to some degree; however, their limitations (eg, high reagent cost, low yields, and potential contaminants) cannot be ignored.^130,131^ In conclusion, a standardized method for the extraction of EVs from different cell sources is lacking and needs to be developed through further research; thus, multiple processes are typically used in combination in practical applications, as shown in Table 1.

While the repair-promoting functions of EVs have been extensively investigated, their clinical applications in periodontitis therapy are still in their infancy.^17^ A human clinical trial was conducted by Beni-Suef University (NCT04270006, Egypt) to evaluate the effect of Exos from ADSCs in the treatment of periodontitis. Ten participants were randomly divided into normal and treatment groups on the basis of their periodontal state. However, the results of this trial have not yet been reported. Another clinical trial, conducted by Xi’an Jiaotong University (ChiCTR1900027140, China), recruited 16 participants from both the normal and treatment groups to investigate the efficacy of autologous DPSC-Exo transplantation in chronic periodontitis. Similarly, the trial results have not been published. However, nonperiodontal clinical trials using MSC-EVs were found in the Cochrane Library database. Most of these trials reported positive therapeutic effects on conditions such as Alzheimer’s disease,^132^ stroke,^133,134^ myocardial infarction,^135^ and skin aging.^136^ Furthermore, several patents have investigated regarding the production and roles of MSC-EVs in periodontal tissue regeneration, as well as their ability to be modified for clinical applications. Most of these patents focus on bone regeneration, which is essential for achieving successful periodontal regeneration. Some drugs have been developed on the basis of MSC-EVs to restore bone defects, as described in Ceioua patents (CN113559124B, CN110433289A, CN115501252B, and CN116350676B). Another critical issue to explore is how to develop a local delivery system for MSC-EVs,^109^ such as mineralized collagen gel (CN114642630B), nanoparticles loaded with resveratrol (CN117398361A) or biotin-avidin systems (CN116271223B).

Interestingly, many studies have highlighted not only the therapeutic effects of EVs in periodontal tissue regeneration but also their roles in the early diagnosis and immunotherapy of periodontitis.^20^ For example, several studies have indicated that EVs and their cargoes can serve as small biomarkers for the early detection of periodontitis.^137,138^ Furthermore, researchers have explored the use of EVs in the development of periodontal vaccines, leveraging the unique properties and functions of EVs.^139^ Although these positive effects are promising, substantial clinical evidence is currently lacking, highlighting an area where further efforts are essential in the future.

Overall, MSC-EVs have good potential in regenerative medicine because of their biocompatibility, low immunogenicity, nanoscale size, engineering versatility, and, notably, capacity to transport diverse bioactive cargoes. Nonetheless, there are still critical challenges that need to be resolved before these materials can be used in clinical treatment. First, achieving the commercialization of EVs presents a critical and challenging hurdle, as large-scale production of high-quality EVs along with a standard or harmonized method for their isolation and purification is needed.^17^ Furthermore, the regeneration of periodontal tissue is a complex process that involves the migration and proliferation of PDLSCs, as well as the reformation of bone, cementum, and vascular structures. However, the fundamental mechanisms governing cementogenesis, angiogenesis, and cellular migration in periodontal regeneration are not yet fully understood,^73,122,126,140^ impeding the realization of complete periodontal regeneration. More importantly, there are very few clinical trials on the use of EVs in periodontitis, with almost no publishable results. Even the most refined rat models of periodontitis cannot fully mimic the intricate conditions of human periodontitis, thereby necessitating additional evidence from clinical trials before clinical application is feasible. However, many unknown risks remain, such as the inability to control its local aggregation or distant dissemination once it enters the body, which may lead to adverse reactions in healthy tissues.^141^ Therefore, advancing the clinical translation of EVs is the direction we need to strive for in the future.

## Conclusion

As discussed above, EVs derived from bacteria and cells exert both pathogenic and pro-repair effects on periodontal tissues, depending on the cargoes inherited from their parental bacteria or cells. BEVs can enrich virulence factors, amplify the immune response and induce heavy bone resorption. The development of effective ways to capture and clear the generated BEVs may help to reshape periodontal homeostasis. The repair-promoting effects of MSC-EVs in the treatment of periodontitis have been heavily explored and indicate that MSC-EVs may be good alternatives for their parental MSCs. Accumulating evidence has revealed the underlying mechanism for the excellent anti-inflammatory, pro-osteogenic/cementogenic/angiogenic, pro-stem cell migratory and proliferative effects of MSC-EVs, owing to their bioactive cargoes. Moreover, the therapeutic performance of MSC-EVs could be further optimized through various methods, such as preconditioning of MSCs, incorporation of bioactive cargoes or fabrication of local delivery systems. The increased therapeutic efficacy of modified MSC-EVs paves the way for the broad application of MSC-EVs for various biomedical applications, with a particular emphasis on periodontitis treatments. Despite the promising potential for the clinical translation of MSC-EVs in the treatment of periodontitis, several major challenges remain to be addressed. Notably, issues such as efficient storage, cost-effective production, and determination of the most appropriate sources for MSC-EVs need resolution. Furthermore, while the combination of MSC-EVs with biomaterials offers promising prospects, the optimal release profiles of MSC-EVs in periodontal defects require further investigation. Additionally, the application of sophisticated bioengineering technologies and advanced materials may limit clinical translation, necessitating careful consideration of costs, particularly as periodontitis is not a life-threatening disease.

## Data Availability

The data underlying this article will be shared upon reasonable request to the corresponding author.
